# FACS-Based Proteomics Enables Profiling of Proteins in Rare Cell Populations

**DOI:** 10.3390/ijms21186557

**Published:** 2020-09-08

**Authors:** Evelyne Maes, Nathalie Cools, Hanny Willems, Geert Baggerman

**Affiliations:** 1 Food & Bio-Based Products, AgResearch Ltd., Lincoln 7674, New Zealand; Evelyne.Maes@agresearch.co.nz; 2 Laboratory of Experimental Hematology, Faculty of Medicine and Health Sciences, Vaccine and Infectious Disease Institute (VaxInfectio), Antwerp University Hospital (UZA), University of Antwerp, 2020 Antwerpen, Belgium; Nathalie.Cools@uza.be; 3 Center for Cell Therapy and Regenerative Medicine, Antwerp University Hospital, 2650 Edegem, Belgium; 4 Centre for Proteomics, University of Antwerp, Groenenborgerlaan 171, 2020 Antwerpen, Belgium; hanny.willems@vito.be; 5 Health Unit, Vlaamse Instelling voor Technologisch Onderzoek (VITO), Boeretang 200, 2400 Mol, Belgium

**Keywords:** FACS, proteomics, cellular heterogeneity

## Abstract

Understanding disease pathology often does not require an overall proteomic analysis of clinical samples but rather the analysis of different, often rare, subpopulations of cells in a heterogeneous mixture of cell types. For the isolation of pre-specified cellular subtypes, fluorescence activated cell sorting (FACS) is commonly used for its ability to isolate the required cell populations with high purity, even of scarce cell types. The proteomic analysis of a limited number of FACS-sorted cells, however, is very challenging as both sample preparation inefficiencies and limits in terms of instrument sensitivity are present. In this study, we used CD14+CD15+ immune cells sorted out of peripheral blood mononuclear cells isolated from whole blood to improve and evaluate FACS-based proteomics. To optimize both the protein extraction protocol and the mass spectrometry (MS) data acquisition method, PBMCs as well as commercialized HeLa digest were used. To reflect the limited number of sorted cells in some clinical samples, different numbers of sorted cells (1000, 5000, 10,000, or 50,000) were used. This allowed comparing protein profiles across samples with limited protein material and provided further insights in the benefits and limitations of using a very limited numbers of cells.

## 1. Introduction

The cellular heterogeneity of human clinical samples represents a major challenge for clinical proteomics, as in many cases the results achieved by the analysis of undefined cell populations might be misleading. In many biomedical and clinical proteomics experiments, for example, the entire tissue of interest is homogenized or tissue slices are processed resulting in an ‘average’ cellular response. These approaches might deliver valuable information but do not allow obtaining molecular information associated with certain cellular subpopulations of interest or diluting relevant effects on protein expression of distinct cell populations in the overall background below the detection limit. In many research questions, the prefractionation of specific cell types is therefore desirable. These days, a wide range of cell isolation techniques are available, with density gradient centrifugation, cell filtering, and immunoaffinity methods as most prominent. Although density gradients are frequently used for the separation of plasma, platelets, peripheral blood mononuclear cells (PBMCs) or erythrocytes from other blood constituents [1], immunoaffinity techniques can be used in both liquid and tissue biopsies and enable enrichment of a wide variety of cell types and organelles [2,3]. This immune-based enrichment of specific cell types is based on antibodies, and their isolation from human in vivo specimens is thus limited to cell populations that have distinct surface markers [4]. The isolation of specific cell types is these days commonly performed by means of flow cytometry. In flow cytometry and fluorescence activated cell sorting (FACS), the heterogenous cell population from a tissue or liquid biopsy is placed in suspension, all particles (including the living cells) are placed in a fluid stream, enter the flow cell in a cell-by-cell way through a nozzle, pass by a set of lasers, and the light scattering and fluorescence signals of each particle passing by are detected. Based on user-defined settings, individual cells can then be collected into homogeneous fractions [5]. This way, FACS can sort cells based on specific, user-defined light scattering and fluorescent characteristics of each cell type and allow the enrichment of even low abundant subpopulations, with high purity. In addition to fluorescent-based cell sorting, enrichment is also possible by means of magnetic activated cell sorting (MACS). In MACS, small paramagnetic beads, instead of fluorescent tags, are coupled to the antibodies, which allows isolation of cells of interest using specialized magnets. Compared to FACS, MACS has the major advantage of ease-of-use [6]. On the other hand, FACS is by definition a cell-by-cell analysis, while MACS analysis is more of a bulk analysis. Therefore, typically, a higher purity of the desired cell population is achieved by FACS. Regardless of the obtained purity, both FACS and MACS can be used to deplete unwanted cells (negative sorting) or to isolate target cell types (positive sorting).

Proteomics analysis of prespecified cell populations can deliver insights into cell-specific functionalities, which is often essential to obtain more knowledge about their specific role. The restricted amounts of in vivo sourced material (e.g., tissue biopsies) combined with the overall cell heterogeneity, however, complicates the overall proteomics procedure as the number of FACS-enriched cells from in vivo samples is often very limited (<50,000 cells). Although several studies exist in which hundreds of proteins are identified out of only 1000 to 10,000 cells, high sensitivity proteomics required to analyse limited amounts of FACS-sorted clinical samples remains challenging.

In the current study, we demonstrate that FACS-based proteomics of a limited number of sorted cells from human clinical samples is feasible. First, we optimized the liquid chromatography (LC) and mass spectrometry (MS) setup for optimized sensitivity using low extraction equivalents of HeLa digest. To demonstrate the potential of this approach for analysis of clinical samples, a subpopulation of CD14+CD15+ cells isolated from whole blood was used in our optimized protocol and four different amounts of cells were analysed and compared.

## 2. Results

In regular shotgun proteomic analyses usually around 1 µg of protein extract is used in order to obtain ‘optimal’ data resulting in thousands of peptide/protein identifications. In some research questions, however, it is unfeasible to obtain 1 µg of starting material. A case in point is the analysis of FACS-sorted cells where in many cases specific cell subtypes are sorted from a heterogeneous mixture of cells, resulting in only a couple of thousand cells. Proteomic analysis of a low number of FACS sorted cells requires an additional optimization step as FACS sheath fluid is incompatible with further LC-MS approaches.

### 2.1. Sample Preparation

To allow efficient protein extraction and sample clean-up ahead of MS analysis, protein samples need to be cleaned. During all our analyses, high recovery of the proteins is required considering the low numbers of cells sorted. In the FACS-sorted samples, the sheath fluid contain salts, PEG, and possibly some other viscosity-altering molecules, making a sample purification step mandatory. Sample preparation steps inevitably lead to analyte loss. When working with large sample quantities, these losses may not be significant (as long as there are no biases towards a specific group of proteins) but when using sorted cells where only a limited number of cells are available, sample loss can be detrimental to the proteomics experiment. Performing a single overnight acetone precipitation, commonly used in proteomics, could not minimize the effect of the non-MS compatible contaminants in the samples. An additional precipitation with ice-cold acetone (−80 °C), however, was very effective to reduce the level of contamination (i.e., loosing/lowering the ions linked to the sheath flow of the FACS instrument) to such an extent that peptides and proteins could be identified and no major chromatographic peaks containing singly charged ions could be detected.

### 2.2. Peptide Separation and Mass Spectrometry Analysis

**Figure 1:**
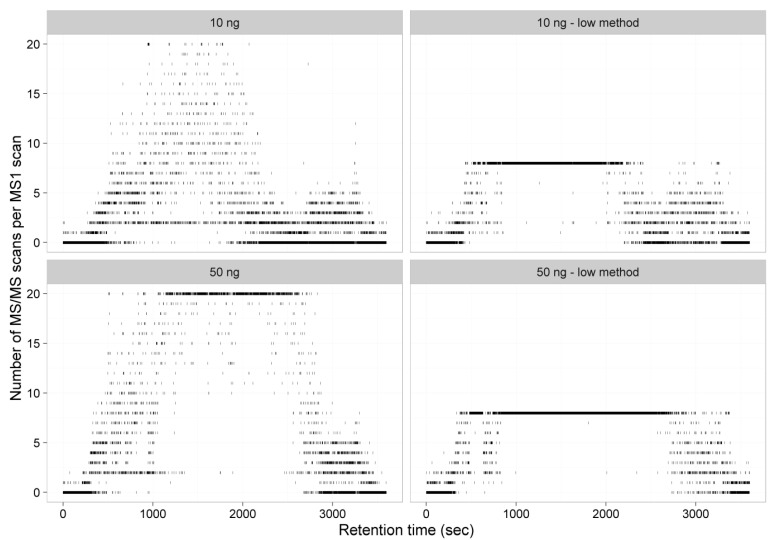
The number of mass spectrometry (MS)/MS scans per MS1 scan in function of the retention time. The samples on the left side are measured using the standard acquisition method. The samples displayed in the righthand panels are measured with the sensitive method, also called the low method.

Next, we evaluated whether a new balance between sensitivity, selectivity, and duty cycle speed of the mass spectrometry analysis was needed to allow a maximum number of high confident peptide identifications in the samples despite the low amount of material. For the purpose of optimization, we analyzed two samples of a commercialized HeLa digest with two different quantities (equivalent to 10 ng or 50 ng protein digest) and performed a standard shotgun analysis’ compared to a ‘sensitive analysis’ based on [7,8]. An overview of our previously used method ‘standard’ vs. the improved method ‘sensitive’ can be found in Table 1. The ‘sensitive’ method has an increased injection time and a higher MS/MS resolution compared to the ‘general’ method at the detriment of the maximum number of MS/MS spectra that can be obtained. As previously described [8], longer cycle times may only be beneficial when peptide concentrations are low and minimal undersampling takes place. A representation of the number of MS/MS per MS1 scans for both methods and protein amounts clearly indicate that, while only a minimal of MS1 scans reach their ‘top 20’ in a general shotgun method of the 10 ng protein equivalent sample (the median number of precursors selected per cycle was 3), almost every MS1 to MS/MS cycle acquires 20 fragmentation spectra in the peptide eluting region of the 50 ng protein equivalent sample. When using the sensitive method, which only selects the top eight precursor ions for fragmentation instead of the top 20 precursors in the general shotgun method, no benefit is found for the 50 ng equivalent sample, as the top eight was almost always reached, hence no improvement to minimize the undersampling issue was found (Figure 1).

Next, we checked whether the higher resolution (which in the orbitrap leads to an increased cycle time) in the ‘sensitive’ method is beneficial for the number of identifications in low concentration samples. Table 2 displays the number of identified non-redundant protein groups, the top proteins (i.e., the protein with the highest sequence coverage amongst the isoforms and fragments identified belonging to that protein) and peptides as well as the number of peptide spectrum matches (PSMs) and MS/MS events. When the number of protein identifications are considered, it is clear that there is a benefit towards the usage of sensitive method when low amounts of sample material is present, as a higher number of identifications are achieved with the same sample. In addition, as the intensity of the MS/MS spectra is higher on average due to longer injection times, the overall % of identified MS/MS spectra is higher in both 10 and 50 ng samples compared to the standard proteomics method.

### 2.3. Low Amount of Proteins and Reproducibility

Next, the ‘sensitive method’ was also tested on a concentration of 1 ng of HeLa protein digest, as some undersampling can still be observed with the 10 ng sample. Additionally, because these amounts are so low, five technical replicates were recorded to see whether our findings are consistent. An overview of the number of identifications are represented in Table 3. Our data indicate that, in our setup, approximately 20 confident protein group identifications are found per sample, and normal inter-sample deviations apply. In total, 29 different protein groups were identified, and 11 protein groups were detected in all five replicates. These groups include high abundant proteins from different histone families, actins, and ubiquitins.

**Figure 2:**
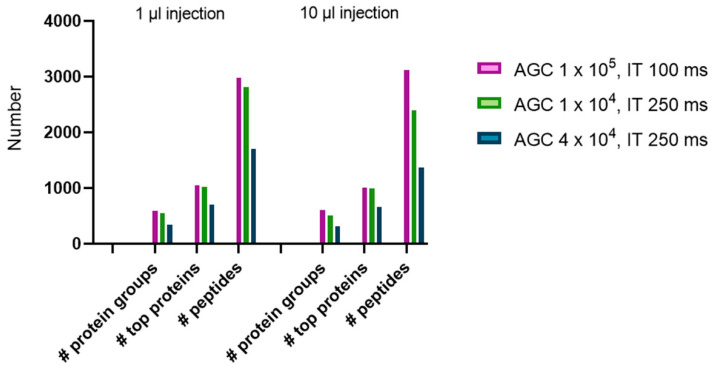
The number of protein groups, top proteins, and peptides based on different injection volumes (1 or 10 µL) and automated gain control (AGC) and injection time (IT) settings.

Subsequently, two other minimal AGC target MS/MS settings are tested to see whether changes in the signal intensity threshold can still increase the number of identifications in these low amount samples. Additionally, we also tested whether an influence of the injection volume of the sample (1 vs. 10 µL injection) is observed. As expected, no influence of the volume of sample injection was observed. However, lowering the AGC threshold has consequences on the number of identifications leading to higher numbers of PSMs (Figure 2).

### 2.4. Application of Method to FACS Sorted Cells

**Figure 3:**
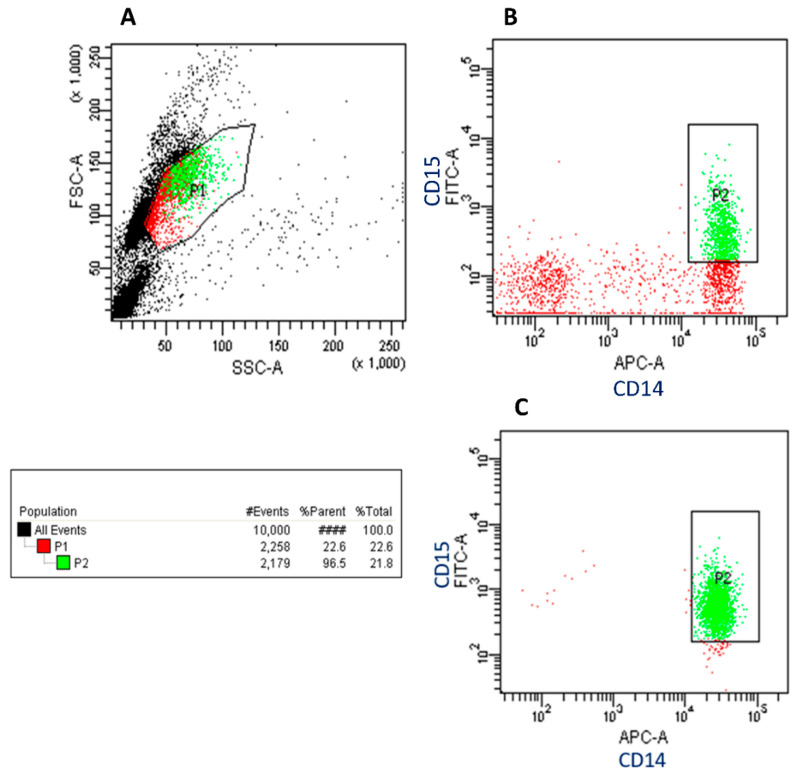
Gating strategy for the analysis of CD14+CD15+ cells from peripheral blood mononuclear cells. (**A**) Plots are gated on forward scatter (FCS) and side scatter (SSC) to define the leukocytes (P1) from cell debris. (**B**) Selection of double positive CD14+CD15+ cell population (P2). (**C**)A post-sort analysis on P2 was performed to determine the purity of the sorted cells. This sorting was confirmed with a purity of 96.5%.

With our optimized proteomics/MS acquisition protocol, we next analyzed FACS sorted pre-specified cell populations. We chose to isolate CD14+CD15+ cells, a population of scarcely present abnormal myeloid cells in whole blood. In the sorting process, different amounts of cells were sorted and collected, respectively: 1000, 5000, 10,000, and 50,000 cells. In Figure 3, the gating strategy for the analysis and sorting of double positive cells (P2) and the purity check of the sorted cells (96.5%) is depicted.

**Figure 4:**
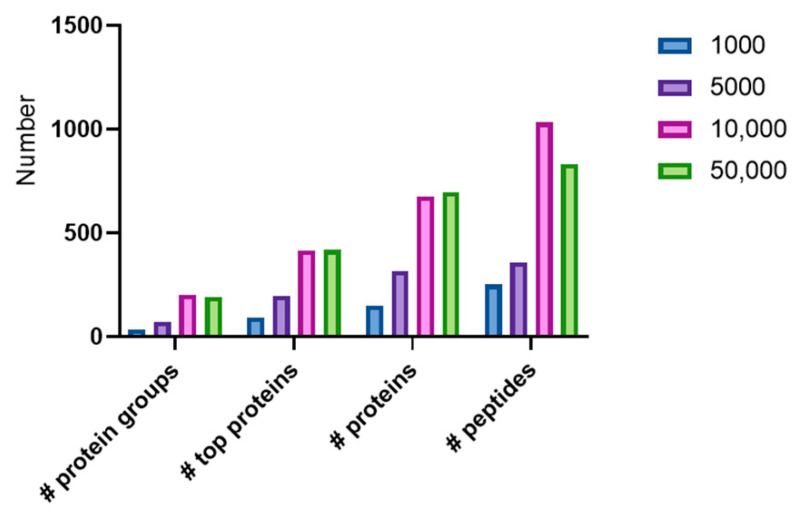
Overview of the number of protein groups, top proteins, proteins, and peptides identified with Peaks X+ in FACS sorted samples on the 1000, 5000, 10,000, and 50,000 cell samples.

Proteomic analysis was performed with the optimized proteomics workflow for FACS sorted samples containing 1000, 5000, 10,000, and 50,000 cells. An overview of the number of protein and peptide identifications in these samples is represented in Figure 4. In this figure, a distinction is made between proteins (each protein that is identified in the database and has a different accession number is counted), top proteins (where proteins with partial sequences are grouped to a top protein), and protein groups which puts all variants, including proteoforms in a similar group. All protein identifications are based on the presence of at least one unique peptide.

While only three protein groups (representing 148 proteins and 83 top proteins) were detected in the sample with 1000 cells, 203 protein groups (representing 677 proteins and 406 top proteins) could be detected in a sample with 10,000 cells. Interestingly, a slight decrease in the number of identifications with 50,000 cells compared to 10,000 cells is seen. While in 10,000 cells 203 protein groups could be identified, only 193 protein groups (compiled from 409 top proteins) were detected in the 50,000 cell sample. This can be explained by the fact that the longer cycle time might be less advantageous for higher protein concentrations. An overview of all the top protein identifications can be found in Supplementary Table S1.

**Figure 5:**
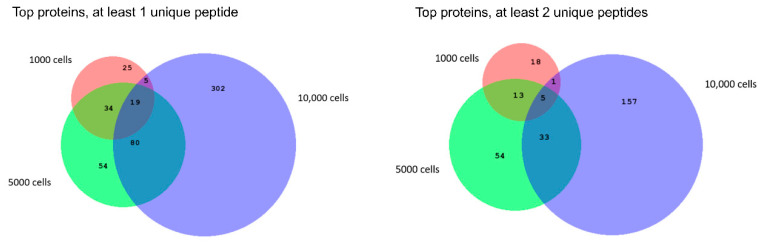
Qualitative proteomic analysis of different amounts of FACS sorted cells. Venn-diagram representing an overlap in the number of top proteins identified between 1000, 5000, and 10,000 cells when either at least one unique peptide per protein is taken into account (left) or when at least two unique peptides per protein are taken into account (right).

Next, we were interested to see how many proteins would be commonly identified between the samples with different amounts of cells (Figure 5). As the sensitive method only proved to be beneficial for the three samples with the lowest number of cells, only a comparison between these three samples was made.

Additionally, a two-fold approach was taken. First, we compared the number of top protein identifications based on the assumption that at least one unique peptide is required per protein hit. This approach allows us to see how extended the protein profile can be in this limited sample amount. With this one peptide per protein rule, 19 top proteins were detected in all three samples, representing only a 4% fraction of the total number of top proteins identified across these samples. Not surprisingly, these 19 proteins represent high abundant proteins such as different classes of histones (i.e., histone H4, histone H2B type 1 and Histone 1.2, Histone H2A type 1), vimentin, elongation factor 1-alpha, actin, and thymosin.

**Figure 6:**
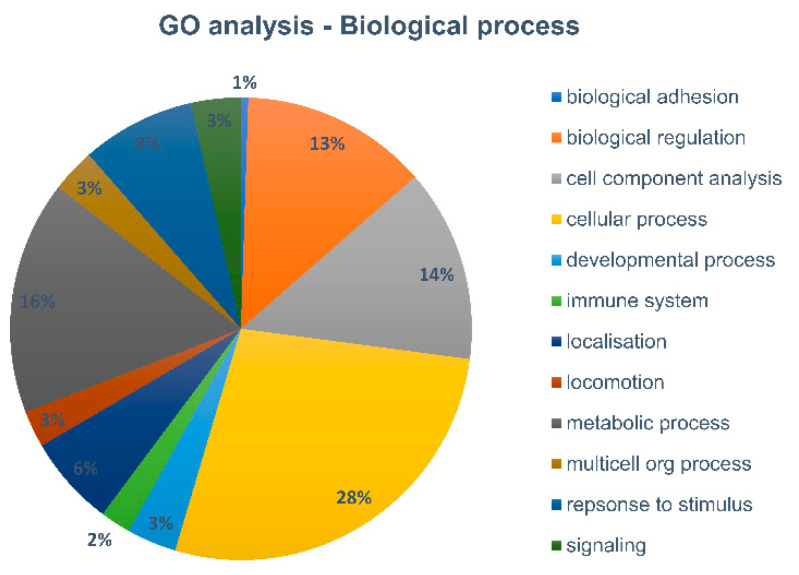
Gene ontology analysis of the 414 top proteins identified in the 10,000 cell sample (based on the one unique peptide per protein rule) and visualized according to the biological process.

Next, the same analysis was performed but only included proteins that were identified with minimal two unique peptides were included in the comparison between the different number of sorted cells to see the potential for future quantitative proteomics work (Figure 6, right panel). Here, only five proteins (Protein S100-A9, Protein S100-A8, Thymosin beta-4, Histone H4, and dermcidin) representing only 2% of the dataset were found in each sample. This clearly indicates that extreme caution should be taken when one would perform quantitative analysis with low sorted cell numbers. However, even with the two unique peptides per protein rule, 196 top proteins were confidently identified in the 10,000 cell sample.

As only a very limited number of proteins, reflecting only high abundant proteins, were identified in the 1000 cells and 5000 cells samples, protein profiling of these low cell numbers might not deliver much insight for downstream proteome analysis. The 10,000 cells sample, on the other hand, has, with around 200 top proteins, has quite a range of proteins identified., To gain further insights in the biological function of these proteins, we performed a gene ontology analysis using PANTHER to classify the identified proteins according to the biological process (Figure 6).

## 3. Discussion

The realistic representation of the in vivo proteome of any subclass of cells in a clinical tissue using proteomic techniques is encumbered by the underlying structure of the heterogeneous microenvironment with spatially intermingled distinct cell types. The analysis of a single cell type can be crucial in the elucidation of cellular functions. To study cell-specific protein expression, it is, therefore, critical to employ methods which allow the sorting of cells according to size, shape, and numerous other cell-specific characteristics/markers. The use of laser capture microdissection (LCM) to isolate individual cells has been implemented in various research projects but the fact that it is a low-throughput technique and that tissue slices are required for this technology, makes it less-widely used [9]. Indeed, as is also the case for histochemistry, with LCM it is difficult to quantitate the levels of expression because: (1) Tissue sections do not contain intact cells, (2) cells frequently overlap, and (3) it is very difficult to discriminate between intermingled positive cells and negative cells for certain membrane-bound proteins [10]. The use of flow cytometry techniques, such as FACS, are very helpful in these cases. Due to the restricted dimensions of clinical tissues available for a wide range of research questions, FACS sorting of a pre-specified type of cells results in many cases only in a limited number of cells (typically between 1000 and 50,000). In addition to the fact that handling a small number of cells can be detrimental for the overall proteome coverage [11], the incompatibility of FACS sheath fluid with proteomics workflows also represents major pitfalls. This makes FACS-based proteomics not straightforward.

Several studies have already been published where proteomics experiments of a minimal number of cells were successful. Highes et al. propose a paramagnetic bead technology to eliminate sample losses during sample preparation of samples limited in quantity [12]. Another machinery is the proteomic reactor, developed by Ethier et al., where a microfluidic device allows the analysis of limited amounts of proteins [13]. A miniaturized LC-MS system for the phosphoproteome analysis of 10,000 cells was proposed by Masuda et al. [14]. Sun et al. analyzed femtograms of protein mixtures by a fast capillary zone electrophoresis-ESI-MS/MS system [15]. The protein content of 100 living cells by direct cell injection was analyzed by the tool developed by [16]. These minimal cell numbers, however, are not achieved with the use of cell sorting technologies.

The combination of antibody-based cell purification and proteomics is also previously described. Di Palma et al. performed a proteomic analysis of 30,000 mice colon stem cells, isolated from intestine tissue via FACS [17]. The enrichment strategy, however, included the sorting of ‘artificial’ green fluorescent protein (GFP)-expressing cells, rather than membrane-expressing protein targeting. Wang et al. simulated circulating tumor cells by adding a minimal amount of cultured MCF-7 tumor cells to whole blood of a healthy volunteer and sorted these MCF-7 out of the blood matrix [18]. Although this already resembles more a real-life situation, still no proteomic analysis is performed of a rare cellular subtype that is isolated via FACS. Additionally, several other studies employ FACS-sorted cells for proteomic analysis, but they all have large quantities of FACS-sorted cells (ca. 1,000,000 cells), which made it possible to perform a buffer exchange immediately after FACS sorting and before the cell pellet is frozen.

To compare protein profiles of rare cell populations, we optimized a workflow that is suitable for low amounts of cells and that allows us to perform LC-MS after FACS sorting. Our optimized method clearly demonstrated the benefit for very low amounts of cellular material, with 10,000 cells as the optimal amount of material. We could demonstrate that the increased cycle time to improve the MS/MS quality of the acquisition method is, however, only favorable in case ultrasensitive proteomics is needed due to low protein concentration.

In this preliminary study, we were able to identify 406 top proteins, representing 203 protein groups from 10,000 CD14+ CD15+ cells in a single LC-MS/MS run. These findings are in line with the FACS sorted experiment described by Di Palma et al., where an 1D-LC experiment of 5000 GFP-sorted cells delivered 380 protein identifications [17]. However, when applying the two unique peptides per protein rule for protein identification, the number of top protein identification drops to 196, demonstrating that for quantitative proteomics purposes, still only a limited number of proteins are detected.

Differences in numbers of protein identification between 10,000 and 1000 cells are still almost 5-fold, indicating that even longer cycle times might be more optimal for 1000 cell samples, and that these very low amounts of cells are still suboptimal for further downstream analysis. However, our 10,000 cell sorted sample demonstrated great potential in the protein profile context. Therefore, a gene ontology analysis was performed to generate an overview of the variety of biological processes the identified proteins are involved in. As expected from immune cells, processes such as response to stimuli or immune response, as well as processes linked to localization and locomotion are detected, amongst the more general cellular and metabolic processes.

In conclusion, these results indicate that we are at the next step in fulfilling the technology requirements towards cell-species-specific proteomics. When proteomics analysis of minimal cell numbers can be achieved, new insights in cellular heterogeneity in clinical tissues might be untangled.

## 4. Materials and Methods

### 4.1. PBMC Isolation and Cell Sorting

Peripheral blood from a healthy volunteer was obtained under consent. The study was conducted in accordance with the Declaration of Helsinki, and the protocol was approved by the Ethics Committee of Antwerp University Hospital (No. 12/7/69). Approximately 20 mL of blood was collected in k_2_EDTA vacutainer blood tubes by venous puncture. Samples were processed within two hours after collection. Peripheral blood mononuclear cells (PBMCs) were isolated by density gradient centrifugation (Ficoll-Paque TM Plus, GE Healthcare, Chalfont St. Giles, UK) according to the manufacturer’s instructions. As PBMCs still represent a heterogeneous mixture of immune cells, we further isolated a specific cell type by fluorescent activated cell sorting (FACS). To reflect the low abundance of a specific type of immune cells in tumor tissues, CD14+CD15+ cells were sorted. Direct immunofluorescence staining was performed with the following fluorochrome-labeled mouse anti-human antibodies: Anti-CD14-allophycocyanin (APC), anti-CD15-fluorescein isothiocyanate (FITC), and anti-CD34-phycoerythrin (PE). For each sample, at least 50,000 events were recorded using a BD FACSAria II flow cytometer (BD Biosciences, Franklin Lakes, NJ, USA). The obtained sorted cell pellets, ranging from 1000 to 50,000 cells were stored at −80 °C until further use.

### 4.2. Sample Preparation of FACS Sorted Cells

The FACS sorted cell pellets were lysed using a RIPA buffer (1×) (Thermo Scientific, Rockford, IL, USA). Depending on the number of cells, different amounts of RIPA buffer were used: An addition of 10 µL RIPA in 1000 and 5000 cells samples, 20 µL with 10,000 cells, and 100 µL with 50,000 cell pellets. Additionally, 0.1% of 1 × HALT phosphatase inhibitor (Thermo Scientific) and 1 × HALT protease inhibitor (Thermo Scientific) were added to each sample. Cell disruption was performed by a 30 s sonication (Branson Sonifier SLPe ultrasonic homogenizer, Labequip, Markham, ON, Canada) of the sample on ice followed by a centrifugation of the samples for 15 min at 14,000× *g* and 4 °C. Afterwards, the pellet was discarded and four volumes of ice-cold acetone (1:4 volumes acetone) were added to each supernatant and incubated at −20 °C overnight. The next day, the samples were centrifuged at 14,000× *g* and 4 °C for 15 min followed by the removal of the acetone. An additional wash with 1 mL of ice-cold acetone (−80 °C) was performed and after centrifugation (14,000× *g*, 4 °C, 10 min), acetone was removed, and the pellet was air-dried for 10 min. Next, the protein pellet was resuspended in 10 µL of a 200 mM TEAB solution.

Next, proteins were reduced by adding 0.5 µL (1000, 5000, and 10,000 cells) or 1 µL (50,000 cells) of 50 mM tris(2-carboxyethyl) phosphine (Thermo Scientific) and incubated for 1 h at 55 °C. Subsequently, cysteines were alkylated by adding 0.5 µL (1000, 5000, or 10,000 cells) or 1 µL (50,000 cells) 375 mM iodoacetamide followed by a 30 min incubation in the dark. Again, the sample is precipitated with acetone by the addition of four volumes of ice-cold acetone per volume of sample and a two-hour incubation at −20 °C. After centrifugation (14,000× *g*, 4 °C, 10 min), acetone was removed, and the pellet was air-dried for 10 min. Next, all protein pellets were resuspended in 10 µL of a 200 mM TEAB solution and trypsin gold (Promega, Madison, WI, USA) was added to a final concentration of 5 ng/µL and incubated overnight at 37 °C. Afterwards, samples were stored at −80 °C until further analysis.

### 4.3. Reversed-Phase Liquid Chromatography and Mass Spectrometry

The peptide mixtures were separated by reversed-phase chromatography on an Easy nLC 1000 (Thermo Scientific) nano-UPLC system using an Acclaim C18 PepMap100 nano-Trap column (75 µm × 2 cm, 3 µm particle size) connected to an Acclaim C18 Pepmap RSLC analytical column (50 µm × 15 cm, 2 µm particle size) (Thermo Scientific). Before loading, the sample was dissolved in 10 µL of mobile phase A (0.1% formic acid in 2% acetonitrile. A linear gradient of mobile phase B (0.1% formic acid in 98% acetonitrile) from 2 %to 35% in 50 min followed by a steep increase to 100% mobile phase B in 5 min flowed by a 5 min period of 100% B was used at a flow rate of 300 nL/min. The nano-LC was coupled online with the mass spectrometer using a stainless-steel nano-bore Emitter (Thermo scientific) coupled to a Nanospray Flex ion source (Thermo Scientific).

The Q Exactive Plus (Thermo Scientific) was used in two different settings: A standard data dependent analysis (DDA) method and a DDA method tuned for higher sensitivity.

The standard shotgun method was set up in MS/MS mode where a full scan spectrum (350–1850 m/z, resolution 70,000) was followed by a maximum of twenty HCD tandem mass spectra in the orbitrap, at a resolution of 17,500. A maximum inject time of 100 ms was set in the full MS, and 80 ms in MS2. The normalized collision energy used was 27 and the minimal AGC target was set at 1.7 × 10^3^. An isolation window of 1.6 m/z and isolation offset of 0.3 m/z was applied. We assigned a dynamic exclusion list of 20 s.

In the sensitive method, a full MS spectrum was acquired at a resolution of 70,000 and a mass range of 350–1850 m/z with a max inject time of 250 ms. Tandem mass spectra (Top 8) were acquired at a resolution of 35,000, with a maximum inject time of 250 ms, a dynamic exclusion of 10 s, an isolation window of 1.6 m/z, isolation offset of 0.3 m/z, and a normalized collision of 27. The minimal AGC target was set at 1 × 10^4^.

### 4.4. Data Analysis

The PEAKS X+ Studio data analysis software package (Bio informatic Solutions Inc., Waterloo, ON, Canada) was used to analyze the LC-MS/MS data. The raw data were refined by a built-in algorithm which allows the association of chimeric spectra. The proteins/peptides were identified with the following parameters: A precursor mass error tolerance of 10 ppm and fragment mass error tolerance of 0.05 Da were allowed, the Uniprot_Homo Sapiens database (v05.2017) was used, and the cRAP database was used as a contaminant database. Trypsin was specified as a digestive enzyme and up to three missed cleavages were allowed. Carbamidomethylation of cysteine was set as fixed modification. Both oxidation (M) and phosphorylation (STY) are chosen as variable modifications. A maximum of three post-translational modifications (PTMs) per sample were permitted. The false discovery rate (FDR) estimation was made based on decoy-fusion. An FDR of <1% at the peptide spectrum match level, and one unique peptide per protein were required to allow confident protein identification. Gene ontology analysis was performed using the PANTHER Classification system [19].

## Figures and Tables

**Table 1:** Settings issued for the standard vs. sensitive acquisition methods.

	Standard	Sensitive
**Full MS**		
Resolution	70,000	70,000
Automatic Gain Control	3.00 × 10^6^	3.00 × 10^6^
Max Inject Time	100 ms	250 ms
Scan range	m/z 350–1800	m/z 350–1800
**MS/MS**		
Resolution	17,500	35,000
Automatic Gain Control	1.00 × 10^5^	1.00 × 10^5^
Max Inject Time	80 ms	250 ms
Top N	20	8
Isolation window	1.6 m/z	1.6 m/z
Isolation offset	0.3 m/z	0.3 m/z
Collision Energy	27	27
Dynamic exclusion	20 s	10 s
Min. AGC target	1.70 × 10^3^	1.00 × 10^4^

**Table 2:** Summary of the number (#) of identified non-redundant protein and peptide groups as well as the number of peptide spectrum matches (PSMs) and MS/MS events acquired with two different acquisition methods and two different protein concentrations.

Name	# Protein Groups	# Top Proteins	# Peptides	# PSMs	# MS/MS	% Spectra Ids
**HeLa_10 ng_standard**	848	1506	4122	5400	13,976	38.6
**HeLa_10 ng_sensitive**	940	1769	4540	5903	9980	59.1
**HeLa_50 ng_standard**	1569	2561	9213	12,855	21,399	60.1
**HeLa_50 ng_sensitive**	1203	2090	6786	10,006	14,195	70.5

**Table 3:** Overview of the number (#) of protein identification groups, top proteins, peptides, and peptide-spectrum matches (PSMs) of five replicates of 1 ng of HeLa protein acquired with the sensitive method.

Name	# Protein Groups	# Top Proteins	# Peptides	# PSMs
Rep 1	20	126	45	128
Rep 2	20	106	50	131
Rep 3	18	107	56	140
Rep 4	17	97	44	110
Rep 5	17	90	40	128

## Supplementary Materials

The following are available online at https://www.mdpi.com/1422-0067/21/18/6557/s1. Table S1: An overview of top protein identifications on the 1000, 5000, 10,000, and 50,000 cell samples.

## Abbreviations

FACS	Fluorescence Activated Cell Sorting
PBMC	Peripheral Blood Mononuclear Cells
MACS	Magnetic Activated Cell Sorting
LC	Liquid Chromatography
MS	Mass Spectrometry
AGC	Automated Gain Control
PSM	Peptide Spectrum Match
LCM	Laser Capture Microdissection
ESI	ElectroSpray Ionization
